# Decision Rightness and Emotional Responses to Abortion in the United States: A Longitudinal Study

**DOI:** 10.1371/journal.pone.0128832

**Published:** 2015-07-08

**Authors:** Corinne H. Rocca, Katrina Kimport, Sarah C. M. Roberts, Heather Gould, John Neuhaus, Diana G. Foster

**Affiliations:** 1 Advancing Standards in Reproductive Health (ANSIRH), Bixby Center for Global Reproductive Health, Department of Obstetrics, Gynecology and Reproductive Sciences, School of Medicine, University of California San Francisco, San Francisco, California, United States of America; 2 Division of Biostatistics, Department of Epidemiology and Biostatistics, University of California San Francisco, San Francisco, California, United States of America; Harvard Medical School, UNITED STATES

## Abstract

**Background:**

Arguments that abortion causes women emotional harm are used to regulate abortion, particularly later procedures, in the United States. However, existing research is inconclusive. We examined women’s emotions and reports of whether the abortion decision was the right one for them over the three years after having an induced abortion.

**Methods:**

We recruited a cohort of women seeking abortions between 2008-2010 at 30 facilities across the United States, selected based on having the latest gestational age limit within 150 miles. Two groups of women (n=667) were followed prospectively for three years: women having first-trimester procedures and women terminating pregnancies within two weeks under facilities’ gestational age limits at the same facilities. Participants completed semiannual phone surveys to assess whether they felt that having the abortion was the right decision for them; negative emotions (regret, anger, guilt, sadness) about the abortion; and positive emotions (relief, happiness). Multivariable mixed-effects models were used to examine changes in each outcome over time, to compare the two groups, and to identify associated factors.

**Results:**

The predicted probability of reporting that abortion was the right decision was over 99% at all time points over three years. Women with more planned pregnancies and who had more difficulty deciding to terminate the pregnancy had lower odds of reporting the abortion was the right decision (aOR=0.71 [0.60, 0.85] and 0.46 [0.36, 0.64], respectively). Both negative and positive emotions declined over time, with no differences between women having procedures near gestational age limits versus first-trimester abortions. Higher perceived community abortion stigma and lower social support were associated with more negative emotions (b=0.45 [0.31, 0.58] and b=-0.61 [-0.93, -0.29], respectively).

**Conclusions:**

Women experienced decreasing emotional intensity over time, and the overwhelming majority of women felt that termination was the right decision for them over three years. Emotional support may be beneficial for women having abortions who report intended pregnancies or difficulty deciding.

## Introduction

Arguments about emotional harms from induced abortion—including decision regret and increasing negative emotions over time—have been leveraged to support abortion regulation in the United States [1–3]. To uphold a 2007 law banning a later abortions, Justice Kennedy of the Supreme Court stated: “While we find no reliable data to measure the phenomenon, it seems unexceptionable to conclude some women come to regret their choice to abort…”[2]. In support of a state-level ban, a researcher testified that abortion “carries greater risk of emotional harm than childbirth”[3]. Arguments about emotional harm have been used to forward parental consent, mandatory ultrasound viewing, and waiting period legislation as well.

Despite these arguments, questions about long-term abortion regret and emotional harm remain unresolved. While research has found that women’s short-term emotions post-abortion can vary substantially—with mixed emotions being common and relief predominating [4–8]—fewer studies have addressed whether decision regret and negative emotions emerge over years post-abortion. Existing longer-term studies suffer from important methodological limitations, including being retrospective and thus vulnerable to selection and recall biases [9, 10]. The few prospective studies have found that most women report positive emotions and satisfaction with the abortion decision years later [6, 7, 11, 12]. But these studies have had mixed results regarding changes in emotions, with some finding decreases in negative emotions over time [6], and others documenting increasing negative emotions and decreasing abortion decision satisfaction [7]. Interpretation is limited by small samples, high attrition, and/or recruitment from single cities or facilities. Additionally, some studies were conducted outside the US or over a decade ago and may not capture the current reality of post-abortion emotions in the US.

Analyses of baseline data from the current study illustrated the importance of differentiating negative emotions from decision regret. Although one-quarter of women experienced primarily negative emotions over one week post-abortion, 95% still felt that the abortion was the right decision [4]. Believing abortion was the wrong decision and experiencing negative emotions are distinct, with the later representing a normal reaction to a significant life event, and the former being an outcome of potential public health concern, yet one that some view as inevitable among some individuals making any decision [13]. While neither construct constitutes a mental disorder, both are important for women’s well-being [10].

Our objective was to investigate how women’s views about the decision to terminate a pregnancy and emotions change over three years. We also compare emotions between women having abortions near facility gestational age limits and women having first-trimester abortions, to elucidate whether emotions differ by gestational age. This is the first study to examine emotions about abortion prospectively in a large, geographically diverse US sample.

## Materials and Methods

### Sample and procedures

We used data from the *Turnaway Study*, a longitudinal study examining the health and socioeconomic consequences of receiving or being denied termination of pregnancy in the US. Between January 2008 and December 2010, 956 women seeking abortions were recruited from 30 facilities across the US. Facilities, described elsewhere, were selected based on having the highest abortion gestational limit within 150 miles [14]. The gestational limits at recruitment facilities ranged from ten weeks through the end of the second trimester due to clinician and facility policy as well as state law. Although abortion has been legal in the US since 1973, law varies greatly by state because individual states may regulate under what circumstances a woman may obtain an abortion, including gestational limits [15].

The primary objective of the *Turnaway Study* is to compare outcomes of women obtaining later abortions to women who were too far along in pregnancy to receive an abortion. In this paper, our main group of interest was women who received abortion within two weeks prior to the facility’s gestational age limit (*Near-Limit Abortion* group). We compared the *Near-Limit* group to women receiving first-trimester procedures at the same facilities (*First-Trimester Abortion* group) to determine whether the experiences of women having later abortions were similar to those of women having procedures in the first trimester, when 92% of US procedures occur [16]. We do not include the third study group, *Turnaways*, comprised of women presenting within three weeks beyond the facility’s gestational age limit who were denied abortions. We could not assess emotions about the abortion or whether women felt the abortion was the right decision among *Turnaways* because the women in this group did not have abortions.

Participant recruitment is described elsewhere [4, 17]. Women presenting for pregnancy termination were eligible if they were English- or Spanish-speaking, ≥15 years old, and had a pregnancy with no known fetal anomalies. Facility staff gave potential participants the informed consent form and connected them by telephone to study staff, who read a consent script, answered questions, and obtained verbal consent over the phone. The participant gave a signed consent form to facility staff, who faxed it to a confidential fax line to the research director. Signed consent forms were sent via FedEx and logged and stored in the research office, separate from participant data or contact information. Administrative procedures required confirmation of paper copy receipt of consent form before interview, which took place at one week after consent. Written parental or guardian consent was obtained for minors seeking abortion in states where parental consent was required for abortion care. In states where parental consent for abortion was not required by law, minors consented to participate in the study themselves. However, in these cases, facility staff first conducted a screening to assess the minor’s ability to consent for herself and her understanding of the potential risks to her in the context of her own life. Because we anticipated that relatively few women would meet *Turnaway* eligibility criteria and to maximize power for primary analyses, we enrolled twice as many participants into the reference group, *Near-Limit*, as into the *Turnaway* or *First-Trimester* groups.

Analyses include data from seven waves of phone interviews, conducted at baseline (approximately eight days after care-seeking) and semiannually thereafter. Baseline interviews assessed sociodemographic characteristics and pregnancy and abortion circumstances; all interviews asked about emotions. Women received $50 gift cards after each interview. Three-year interviews were completed in February 2014.

Overall, 37.5% of eligible women consented to participate, and 85% of those completed baseline interviews (n = 956). Among the *Near-Limit* and *First-Trimester Abortion* groups, 92% completed six-month interviews, and 69% were retained at three years; 93% completed at least one follow-up interview. The final sample size of participants for analyses was 667. Analyses excluded the participants recruited from one site at which all but one *Turnaway* later obtained an abortion elsewhere, because the site did not meet the intended eligibility criterion for the study. We also excluded two *Near-Limit* group and one *First-Trimester* participant who decided not to terminate their pregnancies.

### Ethics Statement

The study, including consent procedures, was approved by the University of California, San Francisco, Committee on Human Research (original approval date: 20 December 2006; study #: 10–00527).

### Measures

#### Outcomes

Decision rightness was assessed at all interviews by asking participants whether, given the situation, the decision to have an abortion was right for them (yes, no, don’t know). For analyses, “don’t know” responses were categorized together with “no” to be conservative. Women were also asked at each interview how much they had felt each of six emotions about the abortion (relief, happiness, regret, guilt, sadness, anger) over the last week (0 = not at all, 1 = a little, 2 = moderately, 3 = quite a bit, 4 = extremely). The emotions examined were drawn from the literature [6–8, 12, 18]. We used responses to the four negative emotions to create a scale (range 0–16; Cronbach’s α = 0.88). Similarly, responses to the two positive emotions were combined into a scale (range 0–8; α = 0.69). To ensure that women responded about the abortion and not the pregnancy itself, these items were preceded by emotions questions regarding the pregnancy. At each follow-up interview, women were asked how often they thought about the pregnancy or abortion (0 = never, 1 = rarely, 2 = sometimes, 3 = fairly often, 4 = all the time).

#### Independent variables

Study group included *Near-Limit* and *First-Trimester*. Time was months from recruitment. *First-Trimester* group-by-time interaction terms were created to assess different emotional time trends between groups.

We included baseline measures describing the circumstances of the pregnancy and abortion. These variables were selected *a priori* as factors hypothesized to affect women’s response to abortion. We used the London Measure of Unplanned Pregnancy to rank pregnancy planning level (range 0–12; α = 0.53) [19]. We assessed difficulty deciding to seek an abortion (0 = very easy to 4 = very difficult). The abortion preference of the man involved in the pregnancy (MIP) was assessed and categorized as: he wanted the abortion; he was not sure; he did not want the abortion; he was not a part of decision-making or did not know about the pregnancy; and, for participants volunteering the response, he left the decision up to the participant. Participants reported whether they were currently in a relationship with the MIP. We examined the two most common reasons for seeking abortion, coded from open-ended responses: not financially prepared and not the right time; responses were not mutually exclusive [20]. To measure perceived abortion stigma, participants indicated how much they would be looked down upon by people in their communities if they knew they had sought an abortion (0 = not at all to 4 = extremely). Social support was assessed using six items derived from the Multidimensional Scale of Perceived Social Support evaluating interpersonal support from family and friends (range 0–4; α = 0.80) [21, 22]. We examined gestational age (weeks) and whether participants had received facility counseling on whether or not to terminate the pregnancy.

Sociodemographic characteristics included age (years), self-reported race/ethnicity (non-Latina white, non-Latina black, Latina, other), prior abortion(s), and number of children raising (0, 1, ≥2). We included participant’s mother’s education as a proxy for socioeconomic status; we did not use income or education due to the young age of many participants. We assessed school/employment status (in school only, employed only, both, neither) and history of depression, using questions from the Composite International Diagnostic Interview [23]. Women who had ever felt sad, depressed, or lost interest in most things for ≥2 weeks, and this seriously interfered with daily activities, were considered to have a history of depression.

### Analyses

To investigate baseline differences between the participant groups, we fit bivariable regression models, including random facility effects to account for the clustering of participants within facilities [24]. Depending on the measurement of the characteristic, we used a linear, logistic, multinomial logistic, or ordinal logistic model.

Our overall approach to longitudinal analyses examining changes in abortion decision rightness and in emotions was mixed-effects regression, including random intercepts for facility and for participant in each model to account for clustering. Random time effects allowing changes in outcomes over time (or trajectories) to differ across participants were included if they significantly improved model fit based on likelihood ratio tests. Similarly, for each model, we sought appropriate functional forms for time by adding quadratic and cubic terms and assessing the statistical significance of the added terms. Interaction terms between study group and time were also included in each model to assess differences in trajectories of outcomes between *Near-Limit* and *First-Trimester* participants. Models also included the *a priori* selected baseline variables thought to affect response to abortion.

Specifically, to assess changes in abortion decision rightness over three years, examine study group differences, and identify associated variables, we used a logistic mixed-effects model with random time effects. Quadratic time terms were not included because they did not improve model fit. We calculated the predicted probability of reporting that abortion was the right decision at a given time using the average individual-level intercepts and trajectories from this model (e.g. random effects equal to zero), with mean-centered covariables equal to zero [25]. We also examined how often women thought about the abortion with a multivariable linear mixed-effects model.

Then, to assess negative emotions, we first used linear mixed-effects regression, including random time effects and quadratic and cubic time terms. Based on this model, we created a dichotomous variable of experiencing an increase of over a point in negative emotions over three years. We then fit logistic mixed-effects models with “increasing trajectory” as the outcome to assess associated factors. A linear mixed-effects model with random time effects and quadratic and cubic time terms was also fit to assess positive emotions.

We performed attrition analyses to examine differential loss-to-follow-up. We conducted sensitivity analyses assessing whether differential enrollment of eligible women across facilities affected our results, repeating analyses including only sites that recruited >50% of eligible women. Also, because the gestational limit for providing abortions fell in or near the first trimester for seven facilities, 14% of *Near-Limit* group participants received abortions in the first-trimester. We thus repeated analyses excluding these seven sites to see if results were consistent. We also repeated analyses including participants from the one excluded recruitment site to see if results were consistent. Stata v.13 was used (College Station, TX, US).

## Results

On average, participants were 25 years old at baseline (Table 1). Approximately one-third were white, one-third black, 21% Latina and 13% other races. Sixty-two percent were raising children, and 14% had a history of depression. Over 53% reported that the decision to seek the abortion was difficult or very difficult. Mean pregnancy planning scores were low, at 2.7 on the 0–12 scale.

**Table 1:** Participant characteristics, by study group: percentages and p-values, *Turnaway Study* (n = 667)

	Near-Limit Abortion	First-Trimester Abortion	p	Total
	(n = 413)	(n = 254)		(n = 667)
**Sociodemographics**				
**Age, mean years** (range: 14–46^a^)	24.9	25.9	0.041	25.3
**Race/ethnicity**				
White	32.0	39.0	0.033	34.6
Black	31.7	31.5		31.6
Latina	21.1	21.3		21.4
Other	15.3	8.3		12.6
**Maternal education**				
<High school	12.4	20.5	0.024	15.4
High school	35.8	35.8		35.8
Some college, technical school	15.0	9.8		13.0
≥College graduate	26.6	28.4		27.3
*Missing*	10.2	5.5		8.4
**Children**				
0	36.4	40.6	0.668	38.0
1	30.3	24.8		28.2
2+	33.3	34.7		33.8
**Prior abortion**	46.5	46.6	0.891	46.6
**School/employment**				
Neither	33.2	23.7	0.013	29.6
In school only	12.6	12.7		12.6
Employed only	40.4	41.5		40.8
Both	13.8	22.1		17.0
**History of depression**	12.8	14.1	0.227	14.1
**Pregnancy Circumstances**				
**Pregnancy planning, mean score** (range:0–12)	2.7	2.6	0.380	2.7
**Difficulty deciding to seek abortion**				
Very easy	10.4	16.9	<0.001	12.9
Somewhat easy	15.7	22.1		18.1
Neither easy nor difficult	15.7	14.6		15.3
Somewhat difficult	27.1	26.8		26.8
Very difficult	31.0	19.7		26.7
**In relationship with MIP**	58.8	58.7	0.986	58.8
**Abortion preference of MIP** (ref: Wanted)				
Wanted	21.1	31.9	0.025	25.2
Not sure	21.6	19.7		18.9
Did not want	21.1	18.9		20.3
Not involved	17.7	16.9		18.9
Left it up to participant	18.5	12.6		16.2
**Abortion Circumstances**				
**Gestational age, mean weeks** (range: 3–29)	19.7	7.6	<0.001	15.1
**Reason for abortion: Financial**	43.6	35.0	0.030	40.3
**Reason for abortion: Not the right time**	34.8	38.6	0.323	36.2
**Perceived abortion stigma**				
Not at all	38.9	41.0	0.412	39.7
A little	14.2	13.7		14.0
Moderately	14.5	16.5		15.2
Quite a bit	13.0	12.5		12.8
Extremely	19.5	16.5		18.3
**Social support, mean score** (range:0–4)	3.2	3.2	0.869	3.2
**Received counseling at facility**	70.1	70.0	0.776	70.0

^a^ One participant aged 14 was recruited before the minimum age was changed to 15.

Compared to the *Near-Limit* group, the *First-Trimester* group was on average older and included a higher proportion of white women. *First-Trimester* participants were more likely to be both in school and employed and had had less difficulty deciding to seek abortion. They were more likely to report that the man involved in the pregnancy had wanted the abortion and were less likely to have sought abortion for financial reasons. By study design, gestational ages were lower in the *First-Trimester* group (mean = 8 weeks) than in the *Near-Limit* group (mean = 20 weeks).

**Fig 1:**
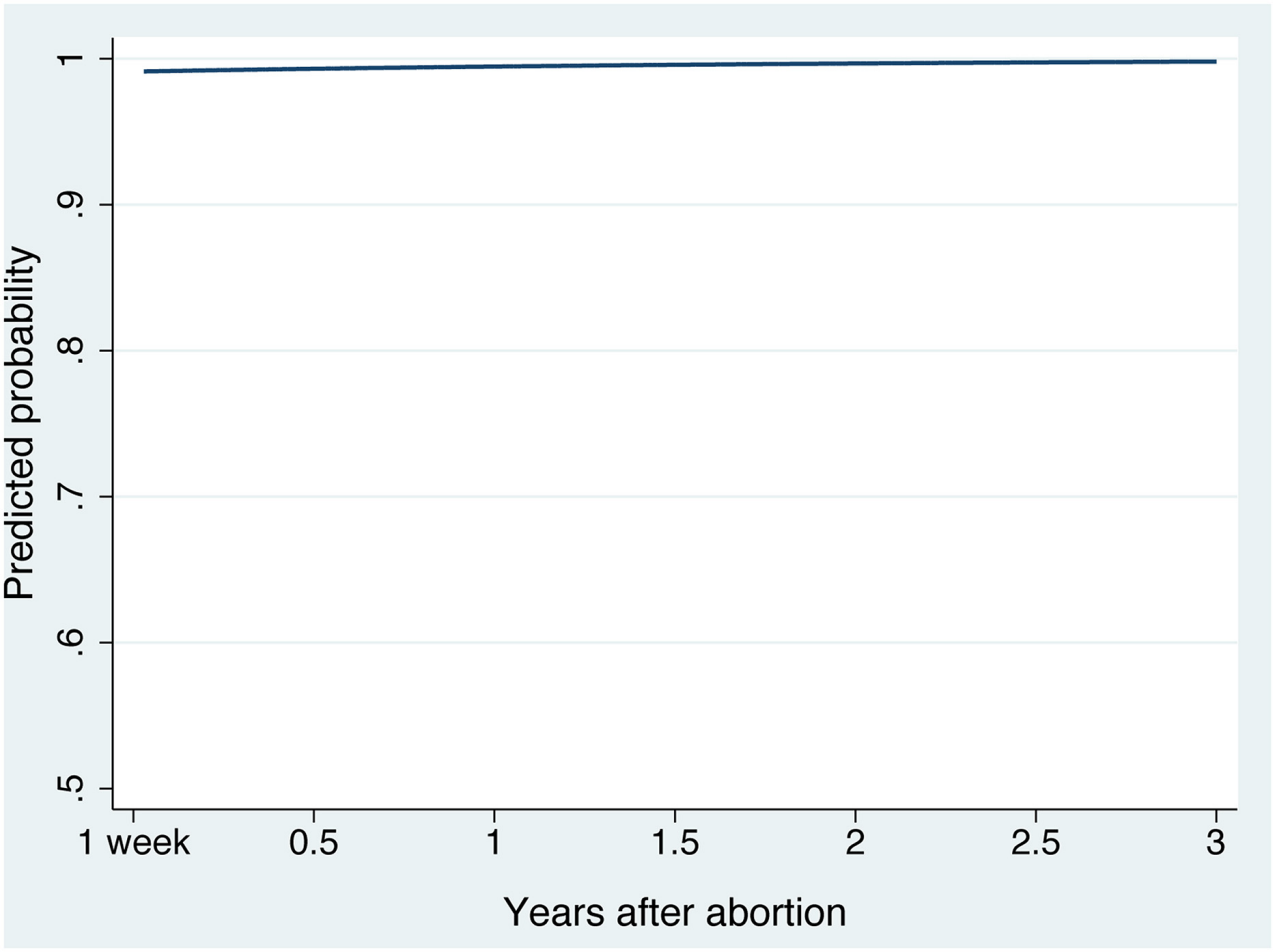
Mean predicted probability of reporting that abortion was the right decision over three years after an abortion. The line represents the trajectory of the average participant (average intercept and slope), based on a multivariable mixed-effects model of reporting that abortion was the right decision, with mean-centered covariables equal to zero.

In crude data, approximately 95% of women completing each follow-up interview reported that having the abortion was the right decision for them. Based on the mixed-effects model, which accounts for attrition and baseline characteristics and allows for individual variation in trajectories over time, the predicted probability of the average participant reporting that the abortion was the right decision was >99% across all times, with an increase over three years (adjusted odds ratio [aOR] = 1.05 per month, 95% confidence interval [CI] [1.00, 1.08]) (Fig 1 and Table 2). Women whose pregnancies had been more planned and who had greater difficulty deciding to seek abortion reported lower levels of decision rightness (aOR = 0.72 [0.60, 0.85] and aOR = 0.48 [0.36, 0.64], respectively), as did Latinas (aOR = 0.31 [0.13, 0.74], versus white). Women both in school and employed at baseline were more likely to report that abortion was right than those neither in school nor employed (aOR = 3.23 [1.06, 9.81]). Women reporting that the man involved in the pregnancy was not a part of the decision-making process had greater feelings of decision rightness than women whose partners did not want or were not sure if they wanted to terminate the pregnancy.

**Table 2:** Abortion decision rightness over 3 years post-abortion: adjusted odds ratios from a multivariable logistic mixed-effects regression model (n = 650).

	Abortion was the right decision	
	Adjusted Odds Ratio	95% CI
**Months**	**1.05** *	1.00–1.08
***First-Trimester* group**	1.58	0.70–3.55
***First-Trimester*** * **months interaction**	0.99	0.95–1.03
**Pregnancy Circumstances**		
**Pregnancy planning score**	**0.72** ***	0.60–0.85
**Difficulty deciding to seek abortion**	**0.48** ***	0.36–0.64
**In relationship with MIP**	0.80	0.41–1.60
**Abortion preference of MIP** (ref: Wanted)		
Not sure	0.58	0.24–1.44
Did not want	0.65	0.26–1.61
Not involved	1.92‡	0.66–5.61
Left decision up to participant	0.86	0.30–2.44
**Abortion Circumstances**		
**Reasons for abortion**		
**Financial**	0.91	0.49–1.71
**Not the right time**	1.01	0.51–2.01
**Perceived abortion stigma**	0.84	0.69–1.02
**Social support**	1.43	0.90–2.30
**Received counseling at facility**	0.82	0.41–1.63
**Sociodemographics**		
**Age**	1.06	1.00–1.14
**Race/ethnicity** (ref: White)		
Black	0.68	0.29–1.59
Latina	**0.31** ** †	0.13–0.74
Other	2.09	0.61–7.09
**Maternal education** (ref: <High school)		
High school	1.63	0.62–4.24
Some college, technical school	0.69	0.23–2.06
≥College graduate	0.83	0.31–2.22
**Children** (ref: 0)		
1	1.05	0.47–2.32
2+	0.86	0.37–2.00
**Prior abortion**	1.23	0.64–2.37
**School/employment** (ref: Neither)		
In school only	1.60	0.54–4.68
Employed only	1.43	0.68–3.03
Both	**3.23** *	1.06–9.81
**History of depression**	0.52	0.22–1.19

***p≤.001.

**p≤.01.

*p≤.05.

†Different from “Other” at p≤.01.

‡Different from “Not sure” and “Did not want” at p≤.05.

Note: Effect estimates are based on 3,758 observations of 650 women (mean 5.8 observations/woman).

Women thought about the abortion less frequently over time (b = -0.019 [-0.023, -0.016] per month), with no differences between study groups (data not shown). At six months post-abortion, participants on average thought about the abortion “sometimes” (mean = 1.8, range 0–4); by three years, they thought about it “rarely” (mean = 1.2, range 0–4).

**Fig 2:**
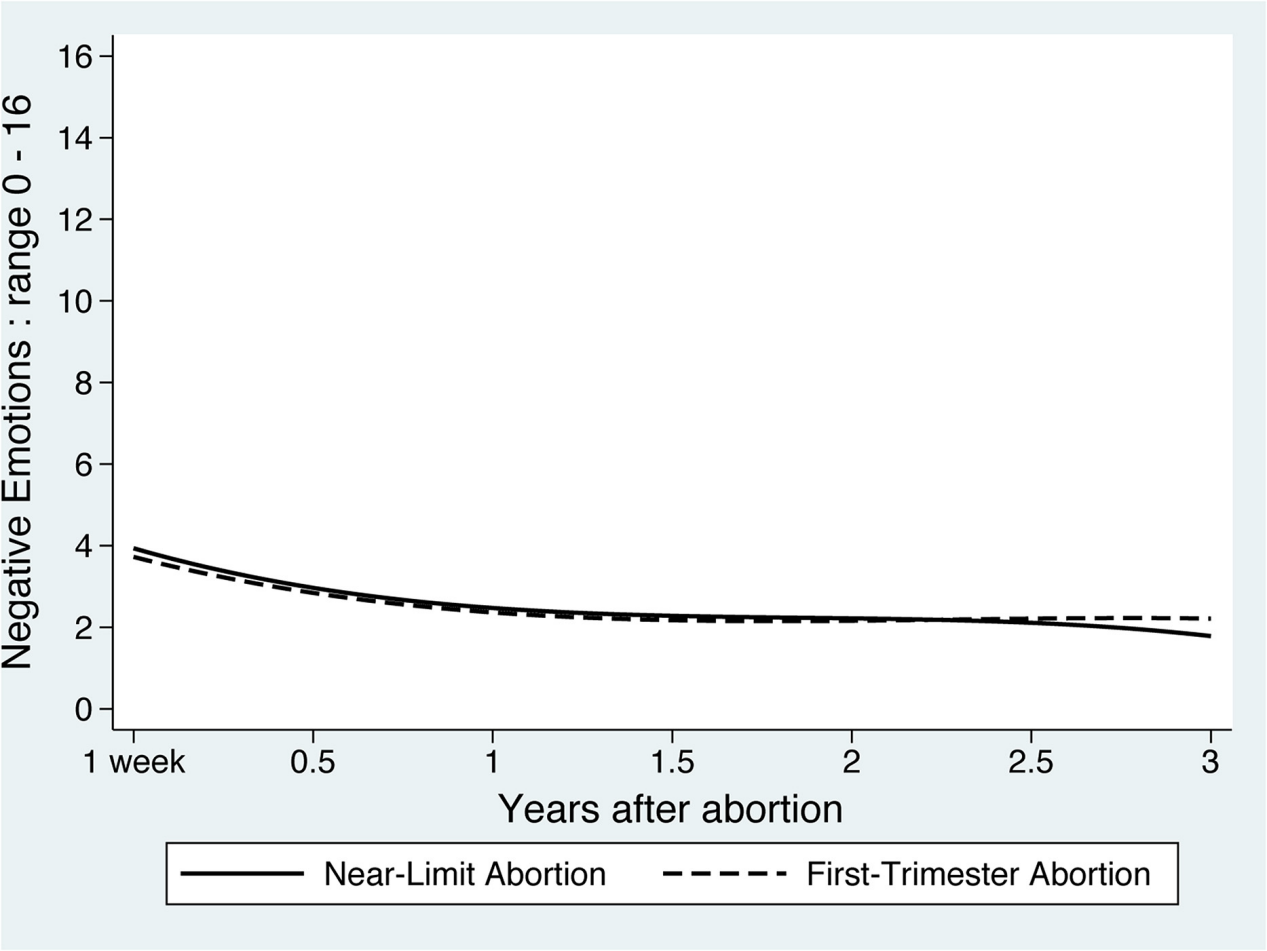
Mean predicted negative emotions scores over three years after an abortion. Lines represent the trajectory of the average participant (average intercept and slope), based on a multivariable mixed-effects model of negative emotions, with mean-centered covariables equal to zero.

The average negative emotions score (range 0–16) among *Near-Limits* declined from 3.9 at baseline to 1.8 at three years (Fig 2 and Table 3). There were no differences in initial level nor change over time in negative emotions for the *First-Trimester* group compared to *Near-Limits* (from 3.7 at baseline to 2.2 at three years).

**Table 3:** Negative emotions (regret, anger, sadness, guilt) over 3 years post-abortion: adjusted coefficients from a multivariable linear mixed-effects model (n = 650).

	Negative Emotions, range: 0–16	
	Adjusted Coefficient	95% CI
**Time**		
**Months**	-0.21***	-0.28 –-0.14
**Months-squared**	0.009***	0.005–0.013
**Months-cubed**	-0.001***	-0.001 –-0.001
**Study Group** (ref: *Near-Limit*)		
***First-Trimester***	-0.21	-0.76–0.34
**Study Group by Time Interactions**		
***First-Trimester*** * **months**	0.02	-0.08–0.13
***First-Trimester*** * **months-squared**	-0.002	-0.009–0.005
***First-Trimester*** * **months-cubed**	0.001	-0.001–0.001
**Pregnancy Circumstances**		
**Pregnancy planning score**	**0.29** ***	0.17–0.42
**Difficulty deciding to seek abortion**	**0.77** ***	0.61–0.92
**In relationship with MIP**	0.05	-0.37–0.47
**Abortion preference of MIP** (ref: Wanted)		
Not sure	0.01	-0.59–0.61
Did not want	0.18	-0.43–0.78
Not involved	0.19	-0.42–0.81
Left decision up to participant	0.12	-0.51–0.76
**Abortion Circumstances**		
**Reasons for abortion**		
**Financial**	0.15	-0.25–0.56
**Not the right time**	-0.18	-0.61–0.24
**Perceived abortion stigma**	**0.45** ***	0.31–0.58
**Social support**	**-0.61** ***	-0.93 –-0.29
**Received counseling at facility**	0.34	-0.09–0.78
**Sociodemographics**		
**Age**	0.01	-0.04–0.05
**Race/ethnicity** (ref: White)		
Black	0.15	-0.38–0.68
Latina	0.47	-0.11–1.06
Other	-0.06	-0.73–0.61
**Maternal education** (ref: <High school)		
High school	0.01	-0.61–0.63
Some college, technical school	0.09	-0.66–0.84
≥College graduate	-0.01	-0.65–0.65
**Children** (ref: 0)		
1	-0.09	-0.60–0.42
2+	0.01	-0.54–0.55
**Prior abortion**	**-0.58** **	-1.00 –-0.16
**School/employment** (ref: Neither)		
In school only	-0.33	-1.03–0.37
Employed only	-0.33	-0.82–0.17
Both	-0.58	-1.23–0.06
**History of depression**	0.55	-0.03–1.14

***p≤.001.

**p≤.01.

*p≤.05.

Note: Effect estimates are based on 3,754 observations of 650 women (mean 5.8 observations/woman).

Over the three years post-abortion, women who had pregnancies that were more planned (b = 0.29 [0.17, 0.42]), who had greater difficulty deciding to seek abortion (b = 0.77 [0.61, 0.92]), and who perceived more community abortion stigma (b = 0.45 [0.31, 0.58]) reported more negative emotions (Table 3). Women with more social support (b = -0.61 [-0.93, -0.29]) and who had had a prior abortion (b = -0.58 [-1.00, -0.16]) reported fewer negative emotions. Approximately 6% of women experienced an increase of at least a point in negative emotions over three years. No baseline factors were significantly associated with having an increasing trajectory of negative emotions (data not shown). Women expressing more negative emotions about the abortion at baseline experienced steeper declines over time (subject-specific slope-intercept correlation = -0.27 [-0.41, -0.12]).

**Fig 3:**
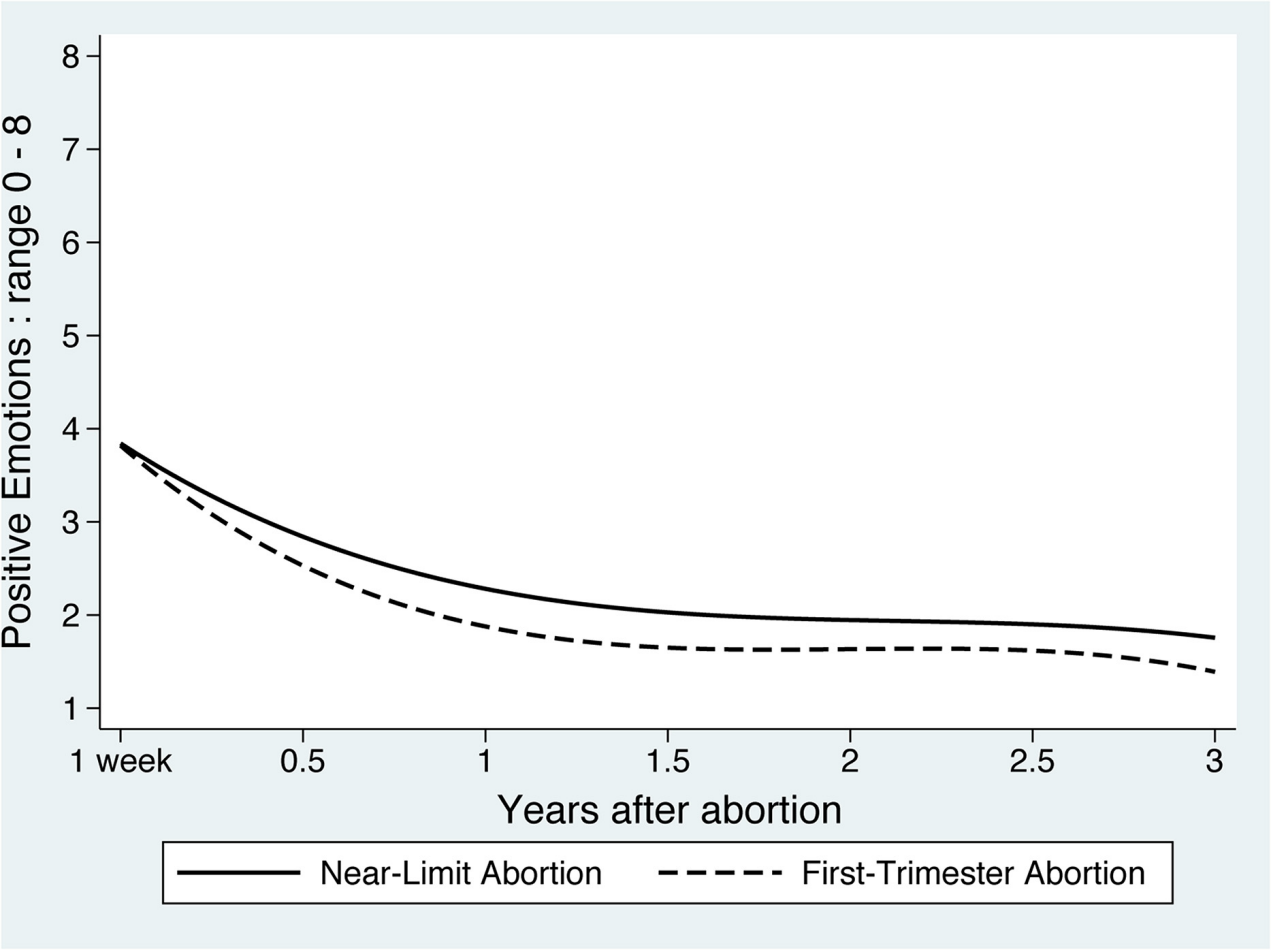
Mean predicted positive emotions scores over three years after an abortion. Lines represent the trajectory of the average participant (average intercept and slope), based on a multivariable mixed-effects model of positive emotions, with mean-centered covariables equal to zero.

For positive emotions about the abortion, average scores (range 0–8) in the *Near-Limit* group declined from 3.8 at baseline to 1.8 at three years (Fig 3, data not shown). Scores for the *First-Trimester* group declined from 3.7 at baseline to 1.4 at three years, reflecting a trajectory no different than for *Near-Limits*. Women with more planned pregnancies (b = -0.09 [-0.17, -0.01]) and who had more difficulty deciding to terminate (b = -0.36 [-0.46, -0.27]) experienced lower levels of happiness and relief. Older women (b = 0.03 [0.01, 0.06] per year) reported more positive emotions, as did black women (b = 0.35 [0.03, 0.68]) and women of other races (b = 0.52 [0.11, 0.93]), compared to white women.

Loss-to-follow-up did not differ by study group, sociodemographic characteristics, nor baseline decision rightness or negative emotions. However, women feeling more relief and happiness at baseline were less likely to be lost (mean score 3.8 for those maintained versus 3.0 for those lost, p = 0.03).

When repeating analyses among sites with >50% participation and, separately, among sites with all *Near-Limit* participants having abortions in the second trimester, results generally remained unchanged, with wider confidence intervals, as expected with smaller sample sizes. The only substantive difference was that, among sites with >50% participation, having a history of depression was significantly associated with lower odds of decision rightness (aOR = 0.25 [0.08–0.78]). Results were unchanged when including participants recruited from the one excluded site.

## Discussion

Arguments that abortion causes women emotional harm, and that women come to regret abortions they decided to have, are used to shape public opinion and advance legislation restricting access to abortion in the US. Existing studies suffer from shortcomings, leaving the question of women’s post-abortion emotions unresolved. Using three years of data from the *Turnaway Study*, we addressed many limitations of prior studies to comprehensively investigate women’s decisional rightness and emotions post-abortion.

Women in this study overwhelmingly felt that the decision was the right one for them: at all time points over three years, 95% of participants reported abortion was the right decision, with the typical participant having a >99% chance of reporting the abortion decision was right for her. Women also experienced reduced emotional intensity over time: the feelings of relief and happiness experienced shortly after the abortion tended to subside, as did negative emotions. Notably, we found no differences in emotional trajectories or decision rightness between women having earlier versus later procedures. Important to women’s reports were social factors surrounding the pregnancy and termination-seeking. Having had difficulty deciding to terminate the pregnancy, and reporting higher pregnancy planning levels, were strongly associated with negative emotions and lower decision rightness, while being in school and working at the time of the pregnancy was associated with far higher feelings of decision rightness. Community stigma and lower social support were associated with negative emotions.

### Strengths and limitations

Analyses included data collected through three years post-abortion. Participant follow-up to five years is ongoing; future analyses will explore how changing circumstances of women’s lives affect feelings about the abortion further into the future.

Because no formal measures of abortion emotions exist, the scales we used may not have validly captured women’s emotions. Although the emotions we examined were similar to those assessed in prior studies [6, 7, 12], they were not necessarily the most relevant aspects of the abortion experience. Relief and happiness may be most relevant directly after an abortion and less relevant over years. In particular, research has found that the positive sentiments women report over time post-abortion included maturity, deeper self-knowledge, and strengthened self-esteem [6]. In addition, social expectations that abortion ought to be emotionally difficult might have led to increased reporting of negative emotions post-abortion [26]. Asking participants biannually about their emotions and how often they thought about the abortion may have led to higher reported levels of all outcomes than otherwise would have existed.

We were unable to assess the effects of continuously measured gestational age on outcomes due to the study design, by which *Near-Limit* participants were recruited within two weeks of facility gestational limits. While this design achieved comparability between the *Near-Limit* and *Turnaway* groups, it resulted in little within-site variation in gestational ages by group. Thus, facility-level factors associated with a facility’s gestational limit, such as state abortion restrictions and community sentiment about abortion, are confounded with individual-level abortion gestational age. That 86% of *Near-Limit* participants had the abortion after the first trimester, and that results did not differ when removing sites with low gestational cut-points, suggest that findings can validly be interpreted as showing a lack of differences in outcomes between women having first-trimester versus later abortions.

Finally, the relatively low participation rate might raise concerns about selection bias. In a review of high-impact public health journals, 63% of prospective studies reported no recruitment information; those that did had participation rates as low as 20% [27]. Another proposed that published participation rates are biased, with studies with lower participation less likely to report participation [28]. 38% enrollment for a five-year study asking women about a stigmatized health service is within the range of other large-scale prospective studies. Importantly, with the exception of being poorer, women in this sample were demographically similar to US women with unintended pregnancies [29]. Also, women experienced a range of emotions at enrollment: approximately two-thirds expressed sadness and over one-third felt some regret [4]. We have no reason to believe that women would select into the study based on how these emotions would evolve over three years.

This study has several features that strengthen the validity of findings. Our use of prospective data helped to reduce recall and selection biases, and we are unaware of other studies prospectively assessing decision rightness and emotions up to three years. Our sample was relatively large, and participants were recruited from diverse geographic locations and across gestational ages, improving generalizability. Only 7% of women were lost-to-follow-up completely after baseline, and our statistical approach accounted for attrition and individual variation in outcomes. Much prior research on post-abortion emotions has been conducted in Europe, where abortion is a viewed differently than in the US; research on US women is an important contribution.

### Interpretation

Results from this study suggest that claims that many women experience abortion decision regret are likely unfounded. The random slope model we fit allowed for individual variability in decision rightness trajectory: some women have lower predicted values of the outcome and others higher values. The typical participant, however, had >99% chance of reporting that the abortion was right for her over three years, and her negative emotions subsided over time. These findings differ from those of the only other large-scale US prospective study, which found that negative emotions increased, and satisfaction with the abortion decision decreased slightly, over two years [7]. Differences in results may be due to differences in outcome measures used, geographic context (one US city in the prior study), time (1993 in the prior study) or attrition (50% in the prior study) [7].

The patterns of emotions found in this study—reduced negative and positive emotions over time after an abortion—indicate a general trend of declining emotional intensity. Various dimensions of psychological welfare, including emotions, are important to women’s well-being after an abortion [10]. Yet no consensus on the meaning of experiencing negative emotions post-abortion exists, and its importance is unclear. Certainly, experiencing feelings of guilt or regret in the short-term after an abortion is not a mental health problem; in fact, such emotions are a normal part of making a life decision that many women in this study found to be difficult [30]. However, increases in negative emotions over time may be indicative of difficulty coping with an abortion, which is a concern for women’s well-being. Our results of declining emotional intensity are consistent with *Turnaway Study* analyses assessing other aspects of psychological well-being, finding steady or improving levels of self-esteem, life satisfaction, stress, social support, stress, substance use, and symptoms of depression and anxiety over time post-abortion [21, 31–34]. The high probability of reporting that the abortion decision was right over all time points is further evidence of emotional coping. Decision regret has been documented among patients undergoing other medical procedures, including sterilization [35], breast cancer treatments [36], and heart surgery [37], as well as among women making other major non-medical life decisions (e.g. marriage, employment), indicating that some level of regret is not unique to abortion[13].

Finally, that higher community abortion stigma was associated with negative emotions—and that having more social support, which may mitigate stigma, was associated with fewer negative emotions—highlights that social context matters for women’s emotions after an abortion [38]. Consistent with prior studies [4, 26, 39, 40], our findings also point to the significance of the decision-making process to post-abortion emotions.

## Conclusions

In the three years after terminating a pregnancy, women tended to cope well emotionally. Women overwhelmingly felt abortion was the right decision in both the short-term and over three years, and the intensity of emotions and frequency of thinking about the abortion declined over time. Yet high coping and resilience were not observed among all individuals: those with more intended pregnancies and difficulty making the abortion decision experienced poorer emotional outcomes after an abortion. Individualized counseling for women having difficulty with the abortion decision might help improve their emotional welfare over time [41]. Efforts to combat stigma may also support the emotional well-being of women terminating pregnancies.
